# Furoquinoline Alkaloids and Methoxyflavones from the Stem Bark of *Melicope madagascariensis* (Baker) T.G. Hartley

**DOI:** 10.1007/s13659-016-0106-6

**Published:** 2016-09-21

**Authors:** Vincent E. Rasamison, Peggy J. Brodie, Emilio F. Merino, Maria B. Cassera, Michel A. Ratsimbason, Stephan Rakotonandrasana, Andriamalala Rakotondrafara, Elie Rafidinarivo, David G. I. Kingston, Harinantenaina L. Rakotondraibe

**Affiliations:** 1 Centre National d’Application de Recherches Pharmaceutiques, B.P. 702, 101 Antananarivo, Madagascar; 2 Department of Chemistry and the Virginia Tech Center for Drug Discovery, M/C 0212, Virginia Tech, Blacksburg, VA 24061 USA; 3 Department of Biochemistry and the Virginia Tech Center for Drug Discovery, M/C 0308, Virginia Tech, Blacksburg, VA 24061 USA; 4 Institut Supérieur de Technologie, B.P. 8122, 101 Antananarivo, Madagascar; 5 Division of Medicinal Chemistry and Pharmacognosy, College of Pharmacy, The Ohio State University, Columbus, OH 43210 USA

**Keywords:** Chemotaxonomy, Furoquinoline alkaloids, Methoxyflavones, Antimalarial activity, Cytotoxicity, *Melicope madagascariensis* (Rutaceae)

## Abstract

**Abstract:**

*Melicope madagascariensis* (Rutaceae) is an endemic plant species of Madagascar that was first classified as a member of the genus *Euodia* J. R. & G. Forst (Rutaceae) under the scientific name *Euodia madagascariensis* Baker. Based on morphological characteristics, Thomas Gordon Hartley taxonomically revised *E. madagascariensis* Baker to be *M. madagascariensis* (Baker) T.G. Hartley. Chemotaxonomical studies have long been used to help the identification and confirmation of taxonomical classification of plant species and botanicals. Aiming to find more evidences to support the taxonomical revision performed on *E. madagascariensis*, we carried out phytochemical investigation of two samples of the plant. Fractionation of the ethanol extracts prepared from two stem bark samples of *M. madagascariensis* (Baker) T.G. Hartley led to the isolation of seven known furoquinoline alkaloids **1**–**7** and two known methoxyflavones **8** and **9**. The presence of furoquinoline alkaloids and methoxyflavones in the title species is in agreement with its taxonomic transfer from *Euodia* to *Melicope*. Antiprotozoal evaluation of the isolated compounds showed that 6-methoxy-7-hydroxydictamnine (heliparvifoline, **3**) showed weak antimalarial activity (IC_50_ = 35 µM) against the chloroquine-resistant strain Dd2 of *Plasmodium falciparum*. Skimmianine (**4**) displayed moderate cytotoxicity with IC_50_ value of 1.5 µM against HT-29 colon cancer cell line whereas 3,5-dihydroxy-3′,4′,7-trimethoxyflavone (**9**) was weakly active in the same assay (IC_50_ = 13.9 µM).

**Graphical Abstract:**

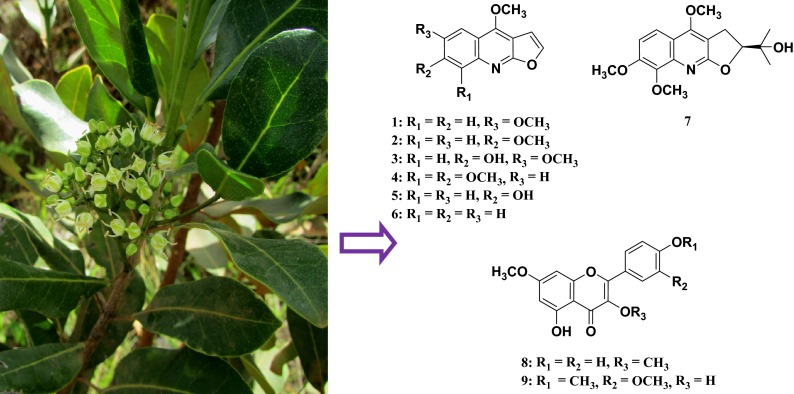

## Introduction

The genus *Melicope* J. R. & G. Forst (Rutaceae) comprises about 235 species distributed in the Madagascar and Mascarenes islands and Indo-Himalaya regions in the Hawaii and Marquesas islands to New Zealand. *Melicope madagascariensis* (Baker) T.G. Hartley is an endemic species to Madagascar, which has long been classified in the genus *Euodia* J. R. & G. Forst (Rutaceae) under the scientific name *Euodia madagascariensis* Baker before being assigned to the genus *Melicope*. This taxonomic revision introduced by Hartley in 2001 was based on morphological characteristics and concerned the other Madagascan plant species that were placed in the genus *Euodia* [1].

*M. madagascariensis* is a tree up to 10–20 m tall which is encountered in the rainforest of Madagascar and is traditionally employed as exhilarating agent, purgative, and in formulations for the treatment of liver, kidney and stomach disorders, bronchitis and mumps [2]. Members of the genus *Melicope* are rich sources of furoquinoline alkaloids, methoxyflavones and acetophenones, some of which have elicited antiplatelet aggregation [3], cytotoxic [4] and antimalarial activities [5]. A previous phytochemical study on *M. madagascariensis* reported the isolation of methoxyflavones [6], a class of compounds that have also been found in *Euodia* species already transferred into *Melicope*. As part of our ongoing projects aiming to characterize secondary metabolites of plants originated from Madagascar rainforest for biological and chemotaxonomical studies, we carried out phytochemical investigation of *M. madagascariensis*, which is one of the eleven *Melicope* species endemic to Madagascar. This paper deals with the isolation of nine known compounds including seven furoquinoline alkaloids **1**–**7** and two methoxyflavones **8** and **9** from two samples of the stem bark of this species. Herein, we discuss the chemotaxonomic significance as well as the antimalarial and cytotoxic activities of the isolated compounds.

## Results and Discussion

A stem bark sample of *M. madagascariensis* was collected from Zahamena, Madagascar. Its ethanol extract (MG250) was first subjected to a modified Kupchan partitioning to give hexane-soluble, chloroform (CHCl_3_)-soluble and aqueous MeOH-soluble extracts. Fractionation of the CHCl_3_-soluble extract by successive open column chromatography over Sephadex LH-20 and silica gel yielded three furoquinoline alkaloids, pteleine (**1**) [7], evolitrine (**2**) [7] and 6-methoxy-7-hydroxydictamnine (heliparvifoline, **3**) [8].

**Fig. 1:**
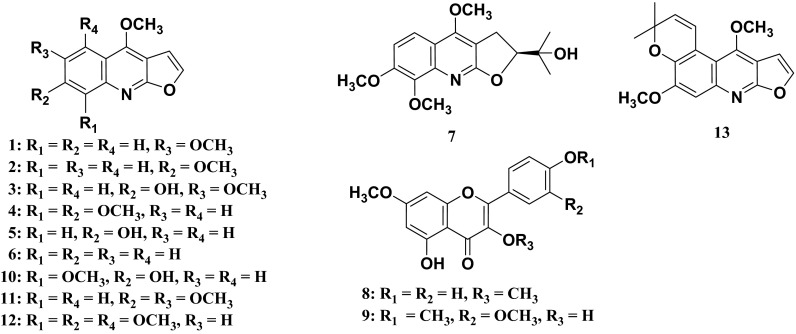
Structures of compounds **1**–**13**

Further phytochemical investigation of the same plant was conducted on a larger collection of stem bark (ST1375) samples collected from Moramanga, Madagascar. Fractionation of the ethanol extract of ST1375 was first carried out by performing selective isolation of alkaloids through conventional acid–base treatment of the CHCl_3_-soluble extract obtained from the same method as above, to finally provide basic and neutral fractions. Subsequent separations of these two fractions by a combination of chromatography techniques (Column Chromatography, HPLC and preparative TLC) yielded four furoquinoline alkaloids identified as: skimmianine (**4**) [9], confusameline (**5**) [10], dictamnine (**6**) [7] and 7,8-dimethoxyplatydesmine (**7**) [11], and two methoxyflavones, kumatakenin (**8**) [12] and 3,5-dihydroxy-3′,4′,7-trimethoxyflavone (**9**) [12]. This is the first report of the isolation of compounds **1**–**9** (Fig. 1) from *M. madagascariensis*. Except the 3,5-dihydroxy-3′,4′,7-trimethoxyflavone (**9**), all the other compounds isolated in the present work have been already encountered in other *Melicope* species, such as *M. semecarpifolia* and *M. pteleifolia* [3, 13].

With the exception of few species including those of the genus *Euodia*, furoquinoline alkaloids are widespread in the Rutaceae family [14]. In particular, a number of furoquinoline alkaloids and derivatives have been reported from species of the genus *Melicope* [3, 15, 16]. The isolation of furoquinoline alkaloids from *M. madagascariensis* chemotaxonomically confirmed the classification of the present plant species in the genus *Melicope* of the Rutaceae family. Furthermore, polyoxygenated flavones (polymethoxy and methylenedioxy) have been considered to be characteristic chemical components of plant species of the genus *Melicope* inside the Rutaceae family [17]. The isolation of furoquinoline alkaloids and methoxylated flavones from the present plant species is in good agreement with its taxonomic reassignment from *Euodia* into *Melicope*. This study added one more species to the *Melicope*-producing methoxyflavones. Whether all of the *Euodia* species containing furoquinoline alkaloids and methoxyflavones should be moved to the genus *Melicope* or not is one of the major questions that can be unfolded from the present investigation. Apart from the morphological and phytochemical contributions, full genome sequencing of all species of the genus to be transferred would complete this taxonomical classification.

The sample MG250 was collected for the International Cooperative Biodiversity Group (ICBG)/Madagascar program that was mainly aimed at searching for antimalarial and antiproliferative agents from natural resources from Madagascar. Evaluation of compounds **1**–**3** for their antimalarial activity against the multidrug-resistant strain Dd2 of *Plasmodium falciparum* showed that only heliparvifoline (**3**) exhibited growth inhibitory effect at relatively high concentration (IC_50_ = 35 µM). Positive control consisted of fosmidomycin (IC_50_ = 0.31 µM). Moreover, in vitro antimalarial activity against the chloroquine-susceptible HB3 (Honduras) and the chloroquine resistant W-2 (Indochina) strains of *P. falciparum* has been previously performed on some Rutaceous furoquinoline alkaloids such as skimmianine (**4**), haplopine (**10**), kokusaginine (**11**), acronycidine (**12**) and acronydine (**13**) [18]. Although the compounds isolated in the present study did not exhibit strong antimalarial activity against the multidrug-resistant strain Dd2 of *Plasmodium falciparum*, the present paper added more data on the antimalarial activity of furoquinoline alkaloids of Rutaceous plants. In addition, compounds **4**–**9** isolated from the sample ST1375 were assayed against the human colorectal adenocarcinoma (HT-29) cell line. As results, compounds **5**–**8** were inactive in this assay while skimmianine (**4**) and 3,5-dihydroxy-3′,4′,7-trimethoxyflavone (**9**) exhibited moderate and weak activity with IC_50_ values of 1.5 µM and 13.9 µM, respectively.

## Conclusions

The current study extends the knowledge about the chemistry of *M. madagascariensis*. The presence of furoquinoline alkaloids and methoxylated flavones in this plant provides support to its re-classification from the genus *Euodia* to *Melicope*. Heliparvifoline (**3**) displayed weak antimalarial activity (IC_50_ = 35 μM) against strain Dd2 of *P. falciparum* whereas skimmianine (**4**) and 3,5-dihydroxy-3′,4′,7-trimethoxyflavone (**9**) were identified as the most cytotoxic constituents against HT-29 colon cancer line.

## Experimental Section

### General Experimental Procedures

^1^H NMR spectra were recorded on Bruker Avance 400 and 500 spectrometers in CDCl_3_ or CD_3_OD with TMS as internal standard. Semi-preparative HPLC was performed on a Shimadzu instrument consisting of LC-20AB pump, SPD-20A Prominence detector and CBM–20Alite system controller. Sephadex LH-20 (Sigma), silica gel 60 (EMD Chemicals, 0.04–0.063 mm) and C18 reversed phase silica gel (EMD Chemicals, 0.04–0.063 mm) were used for column chromatography.

### Antimalarial Assay

The antimalarial assay was carried out at Virginia Tech against the multidrug-resistant strain Dd2 of *P. falciparum* by using the SYBR Green I-based plate assay as previously reported [19].

### Cytotoxicity Assay

Cytotoxicity assay against HT-29 human colon cancer cell line was performed at the College of Pharmacy, The Ohio State University, USA by using a sulforhodamine B reduction assay procedure as described in a previous paper [20].

### Plant Material

The first sample coded MG250 of *M. madagascariensis* stem bark was collected in February 2000 from plants growing in the forest adjacent to the Zahamena National Park. A larger collection of the same plant material designated ST1375 was conducted in November 2009 in the Antsasaka forest of Moramanga. These two collection sites were about 250 km distant from each other in the Atsinanana region of Madagascar. Voucher specimens have been deposited at the herbarium of the Centre National d’Application de Recherches Pharmaceutiques (CNARP), Antananarivo, Madagascar under the codes RJO157 for MG250 and ST1375 B for ST1375.

### Extraction and Isolation

The dried and powdered sample MG250 (300 g) was macerated in EtOH for 48 h to yield 11.5 g of a crude EtOH extract. A portion (1.5 g) was suspended in 90 % aqueous MeOH (150 mL) and extracted with *n*-hexane (3 × 200 mL portions). The aqueous MeOH layer was then diluted to 60 % aqueous MeOH by addition of water before partitioning with CHCl_3_ (3 × 200 mL portions). Finally, the aqueous MeOH phase was vacuum concentrated, suspended in H_2_O (75 mL) and extracted with *n*-BuOH saturated with water (3 × 75 mL portions). Elimination of all the solvents *in vacuo* provided dried *n*-hexane soluble fraction (143.5 mg), CHCl_3_-soluble fraction (538.6 mg), *n*-BuOH-soluble fraction (131 mg) and H_2_O-soluble fraction (630.8 mg). The CHCl_3_-soluble fraction was chromatographed over Sephadex LH-20 column eluted with CH_2_Cl_2_/MeOH, 1:1 and then silica gel column eluted with *n*-hexane/EtOAc, 7:3 to give compounds **1** (0.9 mg), **2** (0.8 mg) and **3** (1.2 mg).

The dried and powdered sample ST1375 (1.3 kg) was extracted as above to afford a crude EtOH extract (65.3 g). A portion (25 g) was liquid–liquid partitioned using same procedures described above. The resulting CHCl_3_ fraction was subjected to a conventional acid–base extraction to afford a basic CHCl_3_-soluble fraction (Fraction A, 343.7 mg) and a neutral CHCl_3_-soluble fraction (Fraction B, 11.2 g). Fraction A was first gel filtered on a Sephadex LH-20 column eluted with *n*-hexane/CH_2_Cl_2_/MeOH, 4:3:3 to give four pooled fractions (A1-A4). Fraction A2 was subjected to a combination of silica gel open column chromatography and HPLC on a semi-preparative C18 column (Purospher column, 5 µm, 25 × 1 cm) or silica gel preparative TLC to furnish **4** (1.4 mg) and **7** (0.9 mg). Fraction A4 (191.4 mg) was rechromatographed over silica gel open column eluted with *n*-hexane/EtOAc mixtures to give **5** (3.1 mg). A part of the fraction B (4 g) was flash chromatographed on silica gel column to furnish six subfractions (B1-B6). Subfraction B2 (171.5 mg) was separated by C18 open column chromatography and then by silica gel preparative TLC with CHCl_3_/MeOH, 50:1 as eluent to furnish **8** (3.4 mg), **9** (2.7 mg) and **6** (1.4 mg).

Structures of isolated compounds were established by the interpretation of their ^1^H NMR spectra and comparison of data obtained with those published in the literature. The ^1^H NMR spectroscopic data of the isolated compounds can be obtained free of charge from the corresponding author.
